# Generalised joint hypermobility and excess knee hyperextension are associated with an increased risk for second ACL injury, but not primary ACL injury, in female football players: A 5‐year follow‐up

**DOI:** 10.1002/ksa.70011

**Published:** 2025-09-09

**Authors:** Anne Fältström, Joanna Kvist, Martin Hägglund

**Affiliations:** ^1^ Region Jönköping County, Rehabilitation Centre Ryhov County Hospital Jönköping Sweden; ^2^ Department of Health, Medicine and Caring Sciences, Unit of Physiotherapy Linköping University Linköping Sweden

**Keywords:** Beighton score, hyperextension, hypermobility, laxity, soccer

## Abstract

**Purpose:**

This study aimed to investigate the association between generalised joint hypermobility, knee hyperextension, knee laxity, and static standing alignment with the risk of anterior cruciate ligament (ACL) injury in a cohort of female football players with an ACL‐reconstructed (ACLR) knee and in knee‐healthy controls.

**Methods:**

We prospectively followed 117 female football players with ACLR (age, mean ± standard deviation, 20 ± 2 years; average 19 ± 9 months after ACLR) and 119 knee‐healthy players (age, 19 ± 3 years) for 5 years. At baseline, all players were assessed for generalised joint hypermobility (Beighton score), knee extension range of motion, knee laxity (KT‐1000, Lachman and pivot shift tests), and static standing alignment (visual assessment graded as varus, valgus or neutral). Log‐binomial regression with risk ratios (RRs) and 95% confidence intervals (CIs) for new ACL injury were calculated. Point biserial and Spearman's rank correlations were used for correlation analysis of baseline anatomical variables.

**Results:**

During the 5‐year follow‐up, 43 ACLR players sustained a second ACL injury (30 re‐ruptures and 13 contralateral ruptures) and 11 knee‐healthy players had an index ACL injury. ACLR players with Beighton score ≥5 (RR, 1.67; 95% CI, 1.04–2.70; *p* = 0.035) and knee hyperextension >5° in the non‐ACL‐reconstructed knee (RR, 1.67; 95% CI, 1.02–2.73; *p* = 0.042) had higher risk of a second ACL injury; knee laxity and static standing alignment were not associated with a second ACL injury (n.s.). No significant associations were seen between baseline variables and index ACL injury in knee‐healthy players (n.s.). There was moderate correlation between KT‐1000 and the Lachman test (*r* = 0.594–0.673), and negligible to moderate correlations between other baseline variables.

**Conclusions:**

Generalised joint hypermobility and knee hyperextension were associated with an increased risk of second ACL injury in female football players with ACLR. Screening for generalised joint hypermobility and knee hyperextension may inform prevention strategies for female football players after ACL injury.

**Level of Evidence:**

Level I.

**Abbreviations**
ACL: anterior cruciate ligament
ACLR: anterior cruciate ligament reconstruction
BMI: body mass index
CI: confidence interval
IQR: interquartile range
RR: risk ratio
SD: standard deviation
SKLR: Swedish Knee Ligament Registry
SPSS: Statistical Package for Social Sciences

## INTRODUCTION

An anterior cruciate ligament (ACL) injury is a severe injury and female football players are at higher risk than males [39]. There is conflicting evidence regarding anatomical factors and association with the risk for a primary or second ACL injury (re‐rupture or contralateral rupture); for instance, generalised joint hypermobility may increase the risk of primary ACL injury [35], but studies show no clear association for a second ACL injury [6, 21, 41]. Similarly, knee hyperextension has been associated with a higher risk of ACL injuries in females [25], although not conclusively [18]. Also, the association between knee laxity [18, 25] and static standing alignment [18, 19] and ACL injury risk remains unclear, with differing reports on its significance.

Understanding how anatomical risk factors relate to ACL injury in female football players is essential for a more thorough and individualised assessment of injury risk and to inform effective prevention strategies. Thus, this study aimed to investigate the association between generalised joint hypermobility, knee hyperextension, knee laxity, and static standing alignment with future ACL injuries for female football players with an ACL‐reconstructed (ACLR) knee and in knee‐healthy female players. A secondary aim was to study the correlation between baseline anatomical variables. The hypothesis was that players with generalised joint hypermobility, excess knee hyperextension, and increased knee laxity, indicated by positive Lachman and pivot shift tests and KT‐1000 side difference in anterior tibial translation, have a higher risk for future ACL injury.

## MATERIALS AND METHODS

### Study design

This was a secondary analysis of a prospective cohort study including 117 female football players with ACLR and 119 knee‐healthy controls. Previous publications with follow‐ups at 2 years [13] and 5–10 years [10, 14] have reported descriptive data on new knee injuries, knee function and activity level outcomes, and functional performance and clinical risk profiles for new or second knee injury [9, 12]. Anatomical factors have not been investigated previously in this cohort except for knee extension included in the clinical risk profile for players with ACLR [12].

### Participants

Female players aged 16–25 years who had undergone primary ACLR 6–36 months prior, were identified from the Swedish Knee Ligament Registry (SKLR). We included currently active football players participating fully in football training at any playing level. Exclusion criteria were additional previous contralateral ACLRs, associated posterior cruciate ligament injury, and/or surgically treated injuries to either the medial or lateral collateral ligament of the knee. This study included 117 players with ACLR (age, mean ± standard deviation [SD], 20 ± 2 years; body mass index [BMI], 23.0 ± 2.6 kg/m^2^); the average time since ACLR was 19 ± 9 months. Autografts were used for ACLR in all players, 114 (97%) with a hamstring graft (58 [51%] with 1‐ to 4‐strand semitendinosus and 56 [49%] with semitendinosus gracilis), 2 (2%) with a patellar tendon graft and 1 (1%) with a quadriceps tendon. The graft size was ≥8 mm in 75 (64%) of cases. Forty‐nine (42%) had a medial and/or lateral meniscus injury (43 [88%] of the meniscus injuries were surgically treated, 17 [40%] of those with meniscal repair) and 11 (9%) had an articular cartilage injury (1 [1%] surgically treated) at the time of the primary ACLR [13]. From 2010 to 2014, femoral drilling was exclusively conducted through a separate medial portal with anatomical position. In our cohort, the predominant method of fixation used in the femur was cortical suspension devices, accounting for 112 (96%) of cases. In the tibia, 69 (59%) utilised intra‐tunnel fixation, while 48 (41%) employed cortical suspension devices.

The knee‐healthy players were recruited by coaches from the same teams as the players with ACLR (matched to playing position and age) and included 119 players (age, mean ± SD 19 ± 3 years; BMI, 22.3 ± 2.2 kg/m^2^). The players participated at various levels from elite to amateur series and were included in the study at the same time point of the football pre‐season (January–April) either in 2013, 2014 or 2015 and followed for 5 years.

All players received written and oral information about the study and signed an informed consent. The study was approved by the Swedish Ethical Review Authority (Dnr 2012/24‐31, 2013/75‐32, 2017/324‐32 and 2020‐01093) and the SKLR board. The previously published data provide a detailed description of the inclusion procedure and descriptive data for the cohort [11, 13].

### Data collection

Baseline assessments of generalised joint hypermobility, knee hyperextension, knee laxity, and static standing alignment were conducted by the same experienced test leader (A.F.) in the same order.

#### Generalised joint hypermobility

Generalised joint hypermobility was evaluated using the Beighton method [3]. The following joints were assessed: passive dorsiflexion and hyperextension in the 5th metacarpophalangeal joints >90° (2 points), passive apposition of the thumbs to the flexor side of the forearm (2 points), >10° hyperextension of the elbows (2 points), >10° hyperextension of the knees (2 points) and active trunk flexion with knees fully extended and palms placed flat against the floor (1 point). The score ranges from 0 to 9; generalised joint hypermobility was defined as ≥5 points [22, 41]. The total score and a cut‐off of ≥5 points versus <5 points were used in the analysis [2]. The Beighton score is a reliable clinical tool with substantial to excellent inter‐ and intrarater reliability [5].

#### Knee extension

**Figure 1:**
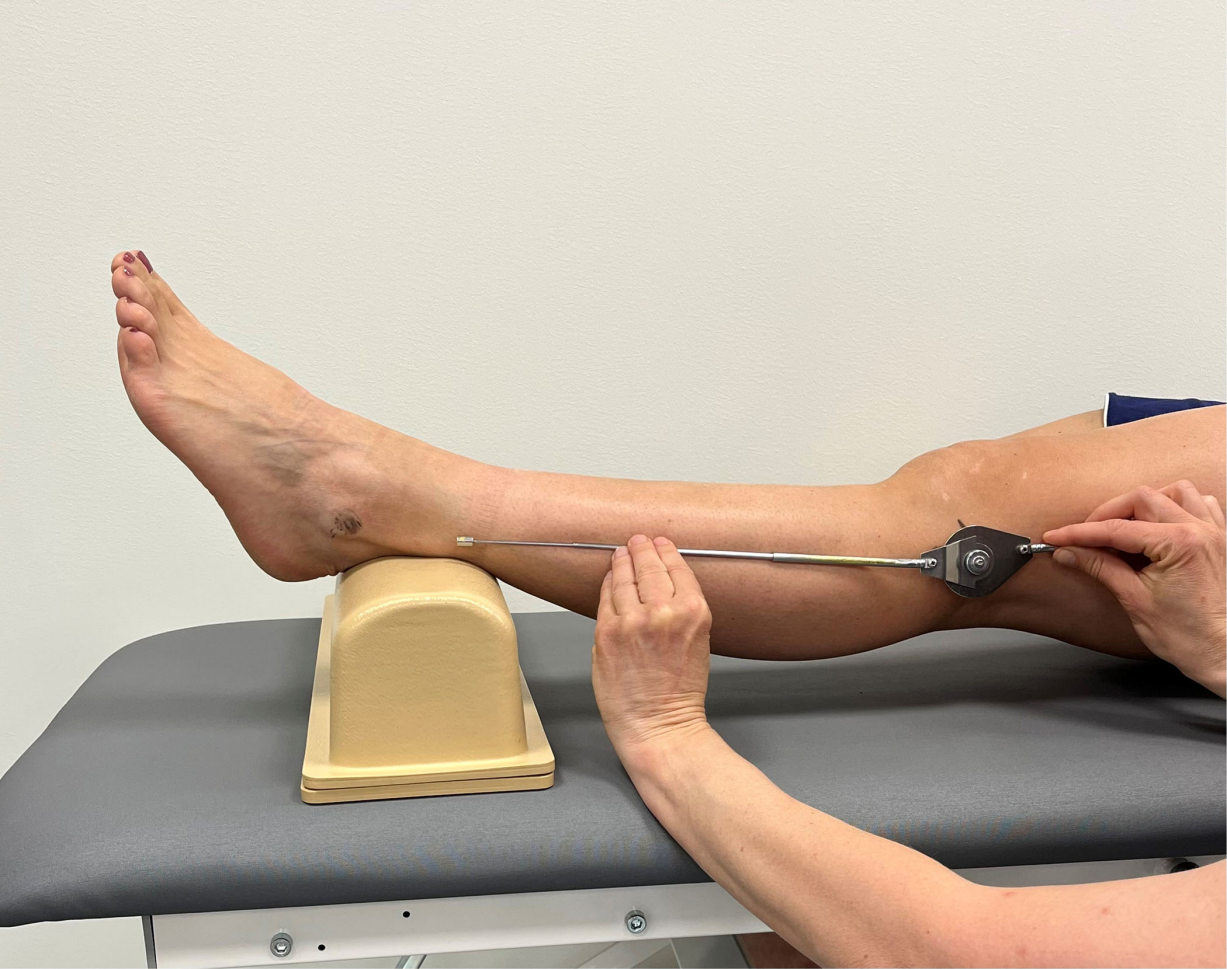
Technique for measuring knee extension with a long arm goniometer with the lateral knee joint line, the most prominent part of the lateral fibular malleolus, and the greater trochanter of the femur as reference points for the goniometer.

Passive knee extension range of motion was measured with a goniometer while the participant was in a supine position with the heel raised on a bolster to ensure that full extension was attained [17] (Figure 1). The intratester reliability for extension metal goniometric measurement of the knee is high (*r* = 0.96) [30]. Knee hyperextension is previously defined as beyond neutral or >5° [15, 16, 18, 25]. For the analysis, a cut‐off of >5° versus ≤5° and four subgroups with no (≤0°), mild (1°–5°), moderate (6°–10°) and severe knee hyperextension (>10°) [36] were used. The analyses included both knees.

#### Knee laxity

The KT‐1000 arthrometer (MEDmetric Corp. San Diego, California) was used to evaluate the amount of anterior translation of the tibia in relation to the femur [7]. The maximum manual test, whereby one hand pulls the tibia forward, was used, and a side‐to‐side difference >2 mm was considered abnormal [1, 7, 17]. For the players with ACLR, the total side difference and a cut‐off of >2 mm versus ≤2 mm in anterior tibial translation were used in the analysis. In addition, knee joint stability was assessed manually with the Lachman test (rated as positive [soft endpoint] or negative [firm endpoint] [24]) and the pivot shift test (rated as positive or negative [11]) and used in the analysis for the players with ACLR. The classification of the Lachman test results into soft and firm endpoint is a reliable and accurate reflection of the status of the ACL [24]. The pivot shift test was rated as positive when the test leader felt a sudden abnormal translation and rotation (subluxation) of the knee joint during flexion or extension [23]. The pivot shift test is the most sensitive test to determine ACL insufficiency [23].

#### Static standing alignment

**Figure 2:**
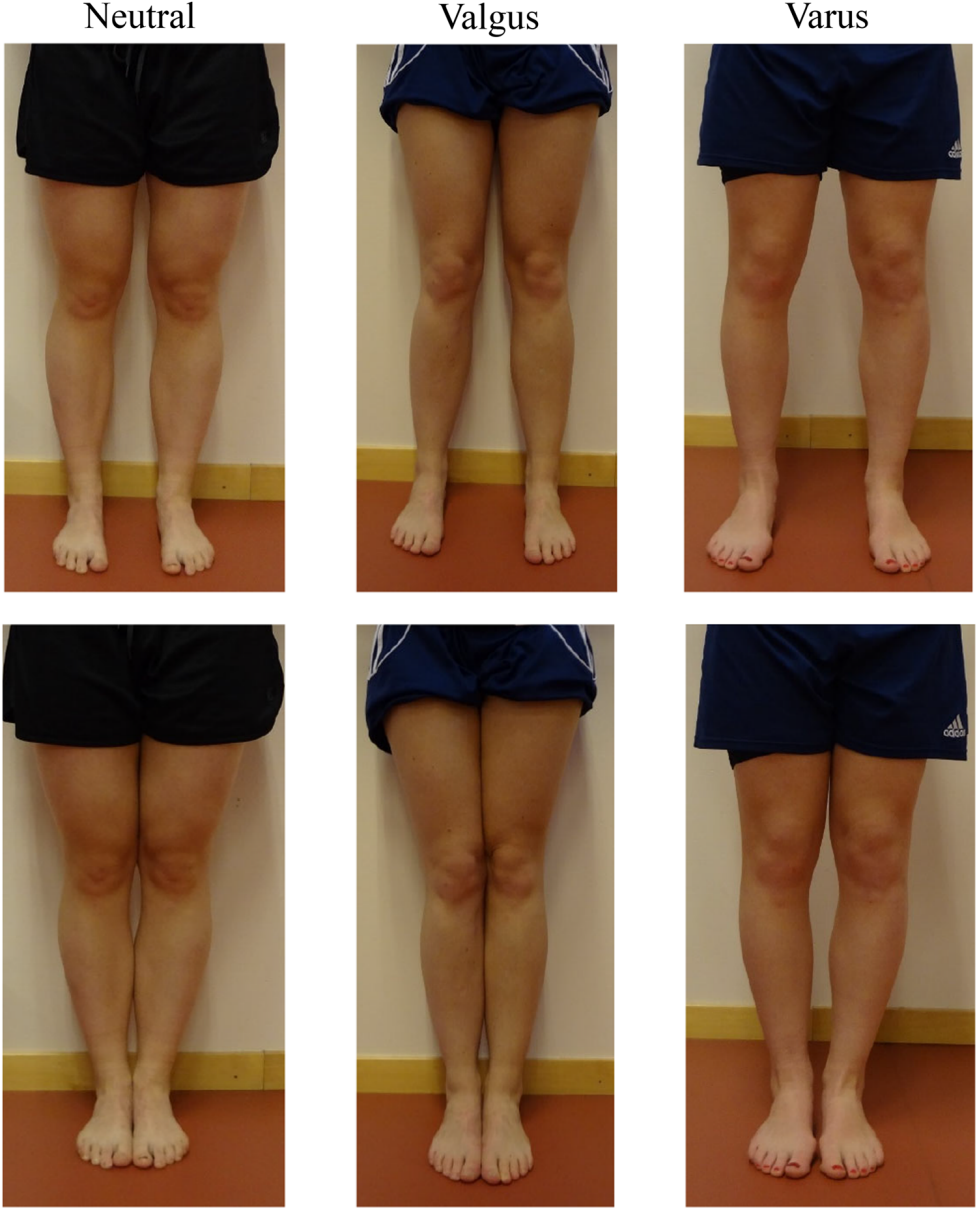
Examples of grading of static standing alignment as neutral, valgus or varus with the feet shoulder‐width apart in a neutral position and with the feet together.

Static standing alignment was visually assessed from a distance of 2 m in the frontal view by the test leader. Participants looked straight ahead, and first stood barefoot with their feet shoulder‐width apart in a neutral position and then with their feet together. During this assessment the test leader estimated the visual angle formed by a line connecting the anterior‐superior iliac spine to the centre of the patella and another line extending from the knee joint centre to the ankle joint centre. They were categorised as having neutral, valgus or varus alignment (Figure 2).

### New ACL injury

Players were followed for 5 years. During this period, players were required to complete a web‐based form six times in the first 2 years–before, during, and after each football season, including a question whether they had sustained any new knee injuries. After 5 years, a question was sent to all players by e‐mail, text message and/or post: ‘Have you injured your knee/ACL again or do you have ACL injuries in both knees?’; fixed response yes or no; and, if applicable, the circumstances and details about the injury. All players suspected of having a severe knee injury without a specific diagnosis were contacted by phone. All new ACL injuries reported by the players were confirmed from the SKLR or medical charts. Complete and partial ruptures described on MRI or arthroscopy procedures were classified as ACL ruptures, particularly when the player reported instability. The response rate was 96% (112 of 117) for the players with ACLR and 95% (113 of 119) for the knee‐healthy players. The 2‐year data (100% response rate) were used in the analyses for the 5 players with ACLR and 6 knee‐healthy players who did not respond to the 5‐year follow‐up.

### Statistical analysis

All statistical analyses were performed using IBM SPSS Statistics for Windows (version 29.0; IBM Corp.; Armonk, NY). Means ± SD or medians and interquartile range (IQR) were calculated for descriptive statistics depending on the data level.

Log‐binomial regression with risk ratios (RRs) and 95% confidence intervals (CIs) were used to analyse associations between generalised joint hypermobility (Beighton score), knee hyperextension, static standing alignment, and new ACL injury during the 5‐year follow‐up (primary for the knee‐healthy players and second [re‐rupture or contralateral ACL injury] for the players with ACLR); a separate analysis was conducted specifically for re‐ruptures. For the players with ACLR, knee laxity measured with KT‐1000, Lachman and pivot shift tests that were rated positive or negative were also included in the analysis.

Correlation between baseline anatomical factors (generalised joint hypermobility, knee hyperextension, knee laxity measured with KT‐1000, Lachman and pivot shift tests [only for the players with ACLR]) and static standing alignment were tested with point biserial and Spearman's rank correlations (Beighton ordinal scale 0–9). The correlations were graded as follows: 0.00–0.10, negligible; 0.10–0.39, weak; 0.40–0.69, moderate; 0.70–0.89, strong; 0.90–1.00, very strong [31]. The level of significance was set at *p* < 0.05.

## RESULTS

Of the 117 players with ACLR at baseline, 43 (37%) sustained a second ACL injury (30 re‐ruptures [70%] and 13 contralateral ruptures [30%]), and 11 of 119 (9%) knee‐healthy players at baseline had an index ACL injury, during the 5‐year follow‐up. Of the 43 new ACL injuries in players with ACLR, 38 (88%) occurred during football, 2 (5%) during skiing, 1 (2%) during handball, and 2 (5%) unknown injury situation. Of the 11 ACL injuries in knee‐healthy players 10 (91%) occurred during football, and 1 (9%) during skiing. Median time from baseline test to new ACL injury was 13.8 months (range, 1–60 months) for the players with ACLR and 18.6 months (range, 1–49 months) for knee‐healthy players. The players were followed until they sustained an ACL injury or for a maximum of 5 years, resulting in an average follow‐up of 4.6 ± 1.5 years.

Players with ACLR with Beighton score ≥5 versus <5 (RR, 1.67; 95% CI, 1.04–2.70; *p* = 0.035) and players with knee hyperextension >5° versus ≤5° in the non‐ACL‐reconstructed knee (RR, 1.67; 95% CI, 1.02–2.73; *p* = 0.042) had significantly higher risk of a second ACL injury. Players with knee hyperextension >5° versus ≤5° in the ACL‐reconstructed knee (RR, 2.04; 95% CI, 1.11–3.76; *p* = 0.022) had a significantly higher risk of re‐rupture. Increased knee laxity and static standing alignment were not associated with a second ACL injury (n.s.) (Table 1). None of the baseline anatomical factors were associated with primary ACL injury in the knee‐healthy players (n.s.) (Table 2).

**Table 1:** Descriptive data and log‐binomial regression analysis with risk ratios and 95% confidence intervals for factors (generalised joint hypermobility, knee extension range of motion, knee laxity, and static standing alignment) associated with a second (re‐rupture or contralateral ACL injury, *n* = 43) and separately for re‐rupture (*n* = 30) for the players with ACL reconstruction (*n* = 117).

Test	Players with ACLR (*n* = 117)						
	Descriptive			Log‐binomial regression analysis			
	No new ACL injury (*n* = 74)	Second ACL injury either knee (*n* = 43)	Re‐rupture ACLR knee (*n* = 30)	Second ACL injury either knee		Re‐rupture ACLR knee	
				RR (95% CI)	*p* value	RR (95% CI)	*p* value
Beighton score							
Median (IQR) (0–9)	1.5 (0–3)	2 (0–5)	2 (0–5)	1.05 (0.96–1.16)	0.295	1.03 (0.91–1.18)	0.612
≥5, *n* (%)	10 (14)	12 (28)	8 (27)	1.67 (1.04–2.70)	**0.035**	1.57 (0.81–3.05)	0.182
Knee extension >5°, *n* (%)							
ACLR limb	23 (31)	19 (44)	16 (53)	1.41 (0.89–2.26)	0.148	2.04 (1.11–3.76)	**0.022**
Uninvolved limb	30 (41)	26 (60)	19 (63)	1.67 (1.02–2.73)	**0.042**	1.88 (0.98–3.60)	0.056
Knee extension subgroups, *n* (%)							
ACLR limb					0.123		0.130
No ≤0°	25 (34)	5 (12)	4 (13)	Reference		Reference	
Mild 1°–5°	26 (35)	19 (44)	10 (33)	2.53 (1.06–6.05)	**0.036**	1.67 (0.58–4.83)	0.347
Moderate 6°–10°	16 (22)	15 (35)	12 (40)	2.90 (1.21–6.99)	**0.017**	2.90 (1.05–8.00)	**0.039**
Severe >10°	7 (9)	4 (9)	4 (13)	2.18 (0.71–6.68)	0.172	2.73 (0.82–9.07)	0.102
Uninvolved limb					0.157		0.150
No ≤0°	17 (23)	1 (2)	1 (3)	Reference		Reference	
Mild 1°–5°	27 (37)	16 (37)	10 (33)	6.70 (0.96–46.79)	0.055	4.89 (0.58–30.34)	0.157
Moderate 6°–10°	18 (24)	16 (37)	10 (33)	8.47 (1.22–58.82)	**0.031**	5.29 (0.74–38.14)	0.098
Severe >10°	12 (16)	10 (23)	9 (30)	8.18 (1.15–58.03)	**0.035**	7.36 (1.03–52.79)	**0.047**
KT‐1000, mm, median (IQR)							
ACLR limb	9 (8–11)	9 (8–11)	10 (8–11)	NA		NA	
Uninvolved limb	7 (6–8)	7 (6–8)	7 (6–8)	NA		NA	
Side difference	2 (0–4)	2 (2–4)	3 (2–4)	1.02 (0.93–1.13)	0.668	1.09 (0.98–1.22)	0.108
>2 mm side difference, *n* (%)	28 (38)	18 (42)	16 (53)	1.11 (0.69–1.79)	0.666	1.76 (0.96–3.26)	0.070
Positive Lachman test, *n* (%)							
ACLR limb	16 (22)	13 (30)	11 (37)	1.32 (0.80–2.16)	0.281	1.76 (0.95–3.24)	0.071
Positive pivot shift, *n* (%)							
ACLR limb	3 (4)	3 (7)	2 (7)	1.39 (0.60–3.21)	0.443	1.32 (0.41–4.28)	0.642
Static standing alignment, *n* (%)					0.816		0.944
Neutral	50 (68)	29 (67)	21 (70)	Reference		Reference	
Varus	2 (3)	2 (5)	1 (3)	0.96 (0.56–1.65)	0.886	0.94 (0.17–5.34)	0.945
Valgus	22 (30)	12 (28)	8 (27)	1.36 (0.49–3.78)	0.553	0.89 (0.44–1.80)	0.736

*Note*: *p* values in bold type are significant.

Abbreviations: ACL, anterior cruciate ligament; ACLR, anterior cruciate ligament reconstruction; CI, confidence interval; IQR, interquartile range; NA, not applicable; RR, relative risk.

**Table 2:** Descriptive data and log‐binomial regression analysis with risk ratios and 95% confidence intervals for factors (generalised joint hypermobility, knee extension range of motion, knee laxity, and static standing alignment) associated with a primary ACL injury (*n* = 11) for the knee‐healthy players (*n* = 119).

Test	Knee‐healthy players (*n* = 119)			
	Descriptive		Log‐binomial regression analysis	
	No ACL injury (*n* = 108)	ACL injury (*n* = 11)	RR (95% CI)	*p* value
Beighton score				
Median (IQR) (0–9)	2 (0–3)	2 (0–3)	0.92 (0.69–1.21)	0.543
≥5, *n* (%)	20 (19)	0 (0)	NA	NA
Knee extension >5°, *n* (%)				
Non‐dominant limb	46 (43)	3 (27)	0.54 (0.15–1.92)	0.338
Dominant limb	40 (37)	3 (27)	0.66 (0.19–2.38)	0.527
Knee extension subgroups, *n* (%)				
Non‐dominant limb				0.715
No ≤0°	22 (20)	2 (18)	Reference	
Mild 1°–5°	40 (37)	6 (55)	1.57 (0.34–7.17)	0.564
Moderate 6°–10°	27 (25)	2 (18)	0.83 (0.13–5.45)	0.844
Severe >10°	19 (18)	1 (9)	0.60 (0.06–6.14)	0.667
Dominant limb				0.844
No ≤0°	23 (21)	2 (18)	Reference	
Mild 1°–5°	45 (42)	6 (55)	1.47 (0.32–6.77)	0.621
Moderate 6°–10°	21 (19)	2 (18)	1.09 (0.17–7.10)	0.931
Severe >10°	19 (18)	1 (9)	0.63 (0.06–6.41)	0.692
KT‐1000, mm, median (IQR)				
Non‐dominant limb	6 (5–7)	6 (6–8)	NA	NA
Dominant limb	6 (5–7)	6 (5–7)	NA	NA
Side difference	0 (0–1)	1 (0–1)	NA	NA
>2 mm side difference, *n* (%)	4 (4)	0 (0)	NA	NA
Static standing alignment, *n* (%)				0.838
Neutral	73 (68)	7 (64)	Reference	
Varus	13 (12)	2 (18)	1.52 (0.35–6.64)	0.575
Valgus	22 (20)	2 (18)	0.95 (0.21–4.29)	0.949

*Note*: The dominant limb is defined as the preferred kicking limb.

Abbreviations: ACL, anterior cruciate ligament; CI, confidence interval; NA, not applicable; RR, relative risk.

For players with ACLR, there was a moderate correlation between the KT‐1000 and the Lachman test (*r* = 0.594–0.673, *p* < 0.05), and weak to moderate correlation between the Beighton score and knee extension (*r* = 0.278–0.484, *p* < 0.05). The other anatomical variables had weak or no correlation (n.s.) (Table 3).

**Table 3:** Correlations between generalised joint hypermobility, knee extension range of motion, knee laxity and static standing alignment for female football players with ACLR.

	Uninvolved limb								
ACLR limb	Beighton score (0–9)	Beighton score cut‐off ≥5	Knee extension, degrees	Knee extension, cut‐off >5°	Knee extension, 4 subcategories	KT‐1000, mm	KT‐1000, side difference >2 mm	Static standing alignment	Lachman test
Beighton score (0–9)			−0.481*	0.388*	0.484*	0.258*	0.047	0.073	
Beighton score ≥5			−0.283*	0.283*	0.311*	0.197*	0.016	−0.028	
Knee extension, degrees	−0.464*	−0.333*				−0.200*	−0.040	−0.141	
Knee extension, >5°	0.365*	0.278*				0.162	−0.001	0.156	
Knee extension, 4 subcategories	0.449*	0.323*				0.194*	0.062	0.115	
KT‐1000, mm	0.259*	0.284*	−0.329*	0.213*	0.265*			0.003	
KT‐1000, side difference >2 mm	0.047	0.016	−0.130	0.054	0.094			−0.062	
Static standing alignment^a^	0.073	−0.028	−0.024	0.183*	0.092	−0.019	−0.062		
Lachman test	0.080	0.078	−0.165	0.066	0.113	0.594*	0.673*	−0.056	
Pivot shift test	0.185*	0.086	−0.120	0.068	0.118	0.269*	0.130	−0.117	0.315*

*Note*: Point biserial and Spearman's rank correlation (Beighton ordinal scale 0–9) were used. Correlation coefficients are presented separately for the ACLR limb and the uninvolved limb.

Abbreviations: ACL, anterior cruciate ligament, ACLR, anterior cruciate ligament reconstruction.

^a^ Visual assessment graded as (1) varus, (2) neutral or (3) valgus.

* *p* < 0.05.

For knee‐healthy players, the anatomical variables had weak or no correlation (Table 4).

**Table 4:** Correlations between generalised joint hypermobility, knee extension range of motion, knee laxity and static standing alignment, for knee‐healthy female football players.

	Dominant limb							
Non‐dominant limb	Beighton score (0–9)	Beighton score cut‐off ≥5	Knee extension, degrees	Knee extension, cut‐off >5°	Knee extension, 4 subcategories	KT‐1000, mm	KT‐1000, side difference >2 mm	Static standing alignment^a^
Beighton score (0–9)			−0.393*	0.297*	0.372*	0.107	0.058	−0.065
Beighton score ≥5			−0.314*	0.270*	0.356*	0.127	−0.084	0.059
Knee extension, degrees	−0.390*	−0.303*				−0.181*	−0.080	0.027
Knee extension, >5°	0.316*	0.263*				0.193*	0.151	−0.039
Knee extension, 4 subcategories	0.382*	0.351*				0.181*	0.081	−0.028
KT‐1000, mm	0.126	0.127	−0.173	0.223*	0.194*			−0.106
KT‐1000, side difference >2 mm	0.058	−0.084	−0.071	0.128	0.070			−0.271*
Static standing alignment^a^	−0.065	0.059	0.013	−0.081	−0.036	−0.106	−0.271*	

*Note*: Point biserial and Spearman's rank correlation (Beighton ordinal scale 0–9) were used. Correlation coefficients are presented separately for the dominant and non‐dominant limbs.

^a^ Visual assessment graded as (1) varus, (2) neutral or (3) valgus.

* *p* < 0.05.

## DISCUSSION

The most important finding of the present study was that the presence of generalised joint hypermobility and knee hyperextension were associated with an increased risk of a second ACL injury in female football players with ACLR. The other anatomical factors, knee laxity and static standing alignment, were not associated with the risk of a second ACL injury. None of the anatomical variables were associated with an index ACL injury in female football players with a healthy knee at baseline.

We found that players with ACLR with generalised joint hypermobility (Beighton score ≥5) had a 67% increased risk of sustaining a second ACL injury compared with players with no generalised joint hypermobility. However, this increased risk did not extend to primary ACL injuries for players who were knee‐healthy at baseline. Other studies have indicated that generalised joint hypermobility increases the risk of primary ACL injury in men [35], as well as the risk of secondary ACL injuries [21, 41]. It has been reported previously that patients with generalised joint hypermobility who undergo ACLR have more than five times higher odds of sustaining a second ACL injury (ipsi‐ or contralateral rupture) within 12 months of return to sport [41]. However, a systematic review found no significant association between generalised joint hypermobility and re‐rupture rates [6]. Furthermore, using a hamstring tendon autograft for ACLR poses more than a fourfold increased risk of re‐rupture in patients with generalised joint hypermobility compared with those with a bone‐patellar tendon‐bone autograft [21]. Hamstring grafts were used in 97% of our cohort. It is theorised that generalised joint hypermobility may create instability during graft maturation, increasing the risk of graft elongation [20]. Even though there are conflicting results regarding the Beighton score and re‐injury risk, it is recommended that the Beighton score should be used to evaluate all patients with ACL injuries to assist in graft selection and rehabilitation because of the high risk of re‐injury, particularly in young athletes [40].

There is about double the risk of a second ACL injury in players with knee hyperextension in their non‐ACL‐reconstructed leg, and similarly for re‐rupture in the ACL‐reconstructed knee when knee hyperextension exceeds 5°. Measuring knee extension in the non‐affected leg is crucial to minimise the impact of the ACLR on knee range of motion. This aligns with previous studies showing that patients with more than 5° of contralateral knee hyperextension with hamstring grafts had a higher rate of graft rupture (15% vs. 3%) within 3 years [15]. In addition, those with >6.5° of knee hyperextension were 14.6 times more likely to experience graft rupture [16]. A similar finding was reported in 183 patients (55% females) with ACLR with auto‐and allografts in a mean follow‐up of 6 years; one‐third of the hypermobile patients, especially with knee hyperextension, sustained a second ACL injury (ipsi‐ or contralateral rupture) [20]. Knee hyperextension has also been linked to graft laxity in recreational athletes, raising concerns about an increased risk of graft failure after ACLR [38].

However, the rate of graft rupture within 5 years was not increased in patients with patellar tendon autografts, even with increased knee hyperextension [4]. The conflicting findings could be because of graft selection, as isolated hamstring grafts are generally not recommended as the first choice in patients with knee hyperextension due to higher failure risks [16]. However, a study that included 5° in the knee hyperextension group did not report any increased revision risk for patients with hamstring grafts [8]. Definitions of knee hyperextension differs, with some studies including 5° [8, 18, 25] and others not [15, 16]. In a previous study, the cut‐off point for knee hyperextension and risk for re‐rupture established by the receiver operating characteristic curve was 6.5° [16]. In our cohort of players with ACLR, the risk for a second ACL injury was increased for players with >0° of extension when analysing with four subgroups [36]. We decided a priori to evaluate the commonly used and clinically relevant cut‐off for knee hyperextension (>5°) in our risk analysis because, in the clinic, knee range of motion is primarily measured in intervals of 5° and considering slight knee hyperextension to be within the normal range [33], thus excluding these players from the knee hyperextension group.

There are conflicting reports on the relationship between knee hyperextension and primary ACL injury. Some studies indicate that females with knee hyperextension who partake in contact sports have a higher risk of ACL injury [25, 37], whereas others found no increased risk [28]. A systematic review identified knee hyperextension as an independent risk factor for ACL injury among high school athletes [27]. Our small sample of knee‐healthy players included only 11 ACL injuries (9%), which may have obscured potential associations.

No increased risk was found for ACL injury related to knee laxity measured with KT‐1000, the Lachman test, and the pivot shift test. Conflicting results exist in previous research; one study indicated a 1.3‐mm increase in knee displacement increased primary ACL injury risk fourfold [25], while another found knee laxity did not significantly affect primary or secondary ACL injury risk in female football and handball players [18]. In addition, KT‐1000 measurements were not significantly different between patients who did or did not sustain a second ACL injury after a mean follow‐up of 6 years [20].

We did not find any association between visually graded static standing alignment and new ACL injury. Static knee alignment, or the Q angle, is a widely used biomechanical measure among clinicians, despite its questionable value [32]. A recent study indicated that visual assessment of lower limb alignment lacks clinical relevance; therefore, physical examination tests and radiographic assessments are recommended for evaluation of alignment [26]. The current literature highlights the limitations of visually graded static standing alignment, including poor reliability, a questionable representation of quadriceps pull, and challenges in translating findings for activities that involve movement [32]. We strived to include screening methods commonly used in clinical settings that do not require any highly specialised equipment, but concur with previous recommendations that visual assessment of static standing alignment provides little value for detecting players at risk for a new ACL injury.

A moderate correlation was found between the KT‐1000 and the Lachman test, and a weak to moderate correlation was observed between the Beighton score and knee extension. In general, correlations between the other anatomical variables included in this study were weak or non‐existent. This aligns with previous reports indicating that generalised joint hypermobility did not correlate with rotatory knee laxity measured by the pivot shift test in ACL‐injured knees [34] or with knee laxity measured by the KT‐2000 in knee‐healthy athletes [29]. Although knee hyperextension is part of the Beighton score, the correlation was only weak to moderate. Knee hyperextension may be an important factor in the Beighton score and for predicting a high‐grade pivot shift [2], but not all patients with knee hyperextension meet the criteria for generalised joint hypermobility [42]. Therefore, the Beighton score, knee hyperextension and knee laxity seem to provide different aspects of knee anatomy (range of motion, sagittal translation, and rotational translation) and association with the risk of new ACL injury.

The strengths of the current study include the homogeneity of the sample regarding age, sex and sport, along with a prospective design and a long‐term (5‐year) follow‐up. All ACL injuries were verified from SKLR or medical charts, which strengthens the validity of the study. Another strength is that all measurements was done by the same experienced test leader, because all measurements performed could differ slightly between assessors. Some limitations should be acknowledged. First, the study sample was limited with few new ACL injury cases, especially for knee‐healthy players. An a priori sample size calculation was not performed for this secondary analysis. Hence, some analyses had limited power to detect associations between anatomical variables and risk of ACL injury, with uncertainties in risk estimates and wide CIs. This must be considered when interpreting the results. Second, we were not aware of the extent of exposure to football matches or training, which is likely the most significant risk factor for new ACL injuries. Finally, we acknowledge that new ACL injuries may be missed, misdiagnosed or not reported after knee trauma at follow‐up.

## CONCLUSION

Generalised joint hypermobility and knee hyperextension were associated with a 67% increased risk of a second ACL injury for female football players with an ACL‐reconstructed knee. These results contribute to a better understanding of the anatomical risk factors for ACL injury. Clinicians should pay particular attention to patients with generalised joint hypermobility and knee hyperextension when assessing the risk of second ACL injury and designing prevention strategies for female football players.

## AUTHOR CONTRIBUTIONS

Anne Fältström, Joanna Kvist, and Martin Hägglund designed the study. Anne Fältström collected and analysed the data. All authors were involved in data interpretation. Anne Fältström wrote the manuscript. All authors read and approved the final manuscript.

## CONFLICT OF INTEREST STATEMENT

The authors declare no conflicts of interest.

## ETHICS STATEMENT

The study was approved by the Swedish Ethical Review Authority (Dnr 2012/24‐31, 2013/75‐32, 2017/324‐32, and 2020‐01093) and followed the principles of the Declaration of Helsinki. All participants were given written information about the study, and signed a written informed consent form before inclusion.

## Data Availability

De‐identified data can be provided on reasonable request.
